# Correlation between *ZBRK1/ZNF350* gene polymorphism and breast cancer

**DOI:** 10.1186/s12920-020-00862-2

**Published:** 2021-01-06

**Authors:** Jun Wu, Alibiati Eni, Eliar Roussuri, Binlin Ma

**Affiliations:** Surgical Department of Breast, Head and Neck Surgery, The Third Clinical Medical College of Xinjiang Medical University (The Affiliated Tumor Hospital), No. 789, Suzhou East Street, Urumqi, 830011 Xinjiang China

**Keywords:** Early-onset breast cancer, *ZBRK1/ZNF350* gene, Single nucleotide polymorphism (SNP)

## Abstract

**Background:**

This study is to explore the relationship between the *ZBRK1/ZNF350* (Zinc finger and BRCA1-interacting protein with KRAB domain-1; also known as zinc-finger protein 350) gene polymorphism and early-onset breast cancer.

**Methods:**

The *ZBRK1/ZNF350* gene exon detection analysis was performed with the direct sequencing and Snapshot methods in 80 cases of breast cancer (aged ≤ 40 years old) and 240 healthy subjects (aged ≤ 40 years old).

**Results:**

Totally 9 sequence variants were detected, including 5 missense mutations and 4 synonymous mutations, located at EXON3, EXON4 and EXON5, respectively. The rs4987241 and rs3764538 variants were published for the first time, while the remaining variants had been reported before. There were significant differences in the frequency distribution of family history between the breast cancer and control groups. Moreover, there were significant differences in the CT genotype frequency at the rs138898320 locus between the breast cancer and healthy control groups. Compared with the carriers of CC wild genotype at rs138898320, the risk of breast cancer was reduced by 88.3% in the CT mutant genotype carriers, with significant difference. In the stratification with no family history, compared with the carriers of CC wild genotype at rs138898320, significant differences were observed for the CT mutant genotype carriers. In the stratification with family history, there was no significant difference in the variation of rs138898320.

**Conclusion:**

The rs138898320 CT mutation genotype of *ZBRK1/ZNF350* may reduce the risk of breast cancer, and the protecting effect would be increased in the stratification with no family history.

*Trial registration* Not applicable.

## Background

Breast cancer is currently the most common malignancy in women worldwide [1]. Generally, breast cancer in young females is more aggressive, with poorer prognosis [2]. About 10%-15% of breast cancer cases are hereditary, which might be related to the mutations of *BRCA1* (Breast cancer susceptibility gene 1) and *BRCA2* (Breast cancer susceptibility gene 2) [3]. However, the clinical significance of some variants is still not clear [4], and the disease may even be partly possible due to the genetic inheritance of multiple susceptible alleles with low penetrance [5].

It is shown that mutations in the *BRCA1* gene can easily cause breast cancer [6]. The *BRCA* gene plays an important role in the homologous recombination mechanism of DNA repairing, and the germline *BRCA1/2* mutations will significantly increase the risk of breast cancer in females [7]. The most common types of disease-causing mutations in *BRCA1/2* are the nonsense mutations and frame-shift mutations. Gene mutations are inherited to offspring in an autosomal dominant manner [8]. The *BRCA1* has a wide range of functions. In addition to participating in the DNA repairing, it also participates in the transcription regulation. However, as a transcription regulator, BRCA1 protein itself cannot directly bind to its downstream genes and a transcription factor with the DNA sequence-specific binding ability is needed to help BRCA1 protein to bind to the downstream genes, thus exerting regulatory functions [9, 10].

*ZBRK1* (Zinc finger and *BRCA1*-interacting protein with KRAB domain-1), also known as zinc-finger protein 350 (*ZNF350*), is a transcriptional suppressor gene, which can specifically recognize a 15-bp DNA sequence, namely GGGxxxCAGxxxTTT (x represents any base) [11, 12]. This sequence exists in the third intron of the downstream gene DNA damage response gene GADD45A regulated by *BRCA1*. BRCA1 binds to GADD45A through ZBRK1/ZNF350 to jointly inhibit the downstream regulatory genes (such as GADD45A), achieving the transcriptional regulation by BRCA1 [12, 13]. The BRCA1/ZBRK1 complex may play a role in the aspartic acid metabolism [14], and the *ZBRK1/ZNF350* gene mutations have been detected in both familial and sporadic breast cancer patients [5, 15]. Moreover, abnormal *ZBRK1/ZNF350* gene expression levels have also been observed in human breast cancer tissues [16]. Therefore, the sequence variation of the *ZBRK1/ZNF350* gene is likely to affect the interaction between its product and BRCA1, thus interfering with the function of the tumor suppressor. Accordingly, *ZBRK1/ZNF350* has been considered to be a new breast cancer susceptibility gene [17]. In this study, the correlation between *ZBRK1/ZNF350* exonic variant and breast cancer was investigated.

## Methods

### Study subjects

Totally 80 Han females with early-onset breast cancer (age of diagnosis ≤ 40 years old), were included in this study, who were confirmed by the pathological diagnosis in the Affiliated Tumor Hospital of Xinjiang Medical University. The blood samples were collected from these patients. None of these patients received radiotherapy or chemotherapy before surgery, and other malignant tumors were excluded. On the other hand, the blood samples were collected from another 240 age-matched healthy Han female subjects in our hospital during the same period. Detailed clinical information, such as the general information and postoperative case report, was obtained. Family hereditary breast cancer must meet the following conditions: (1) Early-onset breast cancer (age of diagnosis ≤ 40 years old); (2) having a family history of breast cancer (at least one of the primary and secondary family members suffering from breast cancer); (3) pathologically diagnosed as breast cancer; and (4) with an expert in breast cancer assigned to evaluate medical history. All enrolled patients signed the informed consent. This study was approved by the Ethics Committee of the Affiliated Tumor Hospital of Xinjiang Medical University.

### Sample storage and DNA extraction

Totally 5 ml peripheral blood was collected, followed by the EDTA anticoagulation, which was stored at—80 °C. DNA extraction was performed with the DNA extraction kits and enzymes, according to the manufacturers’ instructions. The genomic DNA was extracted from the peripheral blood samples with the phenol/chloroform method, and the concentration and purity were determined with the Biophotometer. Totally 1 μl extracted DNA was subjected to the assessment with 1% agarose electrophoresis, and the concentration was diluted down to 5–10 ng/μl.

### DNA sequencing for the case group

The reference sequence used was from the gene database of NCBI (*ZNF350* zinc finger protein 350 [Homo sapiens (human)]; Gene ID: 59,348; https://www.ncbi.nlm.nih.gov/gene/59348). Totally 11 primers were designed and amplified, covering the 2-kb 5′-promoter of the *ZBRK1/ZNF350* gene and gene coding sequence. The primers were designed with Primer3 online software, which were synthesized by the Shanghai Tianhao Biotechnology Co., Ltd., Shanghai, China, with the following sequences: P1, forward 5′-TAGGAAGAGGAGTGGCGTGAA-3′ and reverse 5′-TGCTTCTAAACACAACATTTAAGAAA-3′; P2, forward 5′-AATTCAGGGTGACGAAGTGGT-3′ and reverse 5′-GGAAGAGCAGAGCCAGAATTG-3′; P3, forward 5′-TGTGGGTCATCCTTTACTCTCCT-3′ and reverse 5′-GAATGAATTGGAAATGGAAAAGGT-3′; P4, forward 5′-GTGGGTGTCTGCCTACCTGAT-3′ and reverse 5′-CTCCCAAACCTTCCACTGAGA-3′; P5, forward 5′-TGAGTGCCTAGCAGCAGTGAC-3′ and reverse 5′-TGCATGGTGTCACAAAATTCAT-3′; P6, forward 5′-TGCACACAGCCTGTAGAGTGG-3′ and reverse 5′-ATCTGTAACATCAAGGTTTTTCTCTT-3′; P7, forward 5′-AAGAGAAAAACCTTGATGTTACAGAT-3′ and reverse 5′-CTCACACTGGGAGACAGATGAG-3′; P8, forward 5′-CACAACAAACTTTTACCCCAAATC-3′ and reverse 5′-GAAAGGCTTTGCCACATTCAG-3′; P9, forward 5′-GGGGACTCCTTTCTTCATGCT-3′ and reverse 5′-TTCCACATTCACTGCACACAA-3′; P10, forward 5′-TGAATGTGGCAAAGCCTTTCT-3′ and reverse 5′-CACATCTGACCACTGGCTGTC-3′; and P11, forward 5′-CACACAAGGGAGAAACAAGAGG-3′ and reverse 5′-CCACTTAAAAGTACTTGGGCTTCC-3′. Accordingly, a PCR reaction system was established. The obtained PCR products were subjected to the sequencing analysis with the Big Dye kit (version 3.1; ABI, Grand Island, NY, USA), which was loaded on the ABI3130. The sequencing file was analyzed with the Polyphred software.

### Snapshot typing for the control group

Based on the sequencing results of the case group, the target sites were screened according to the tag site mutual substitution principle and the high frequency sites. The following sites were selected for detection: rs4986771, rs138898320, rs4987241, and rs2278415. For the multiplex PCR reaction, the primers were designed to amplify the fragments containing the above 4 sites, and 4 extension primers adjacent to the SNP sites were designed for the single base extension. Primers were designed using the online Primer3 software. The PCR products were obtained with the HotStarTaq multiplex PCR (203,203; Qiagen). Then the PCR products were purified by the shrimp alkaline enzyme (SAP) (M9910; Promega, Madison, WI, USA) and exonuclease I (EXO I) (X40520K; Epicentre), and the extension was performed with the SNaPshot Multiplex kit (ABI). The extension products were purified with the SAP, which were then loaded on the ABI3730xl. The data were obtained, and the SNP typing was analyzed with the GeneMapper 4.1 (Applied biosystems).

### Statistical analysis

The SPSS 20.0 software was used for statistical analysis. The χ^2^ test was used to compare the counting data (expressed as n(%)) between the case and control groups, and the t-test was used to compare the measurement data (represented by mean ± SD) between the case and control groups. The correlation between the *ZBEK1* gene polymorphism and the case or control group was tested by the χ^2^ test. The binary Logistic regression was used to analyze the *ZBRK1/ZNF350* exon genotypes and breast cancer susceptibility, as well as the OR and confidence interval (95%CI). *P* < 0.05 was considered statistically significant.

## Results

### ZBRK1/ZNF350 gene mutation detection

The *ZBRK1/ZNF350* gene mutation detection was performed for the case and control groups. For the case group, the mutations in the exon region of the *ZBRK1/ZNF350* gene were detected with the direct sequencing method, and totally 9 sequence variants were detected (Table 1), including 5 missense mutations and 4 synonymous mutations located in EXON3, EXON4 and EXON5. These 5 missense mutations were as follows: rs4987241 (c.111G > A, EXON3); rs2278420 (c.197 T > C, EXON4); rs2278415 (c.1503A > T, EXON5); rs4986771 (c.1414 T > C, EXON5); and rs138898320 (c.100C > T, EXON3). These 4 synonymous mutations were as follows: rs4986773 (c.105 T > C, EXON3); rs4988334 (c.708 T > C, EXON5); rs3764538 (c.1119C > A, EXON5); and rs4986772 (c.1155A > C, EXON5). On the other hand, for the control group, according to the sequencing results for the case group, the sequence variants were screened based on the tag site mutual substitution principle and the high frequency sites. The following sites were selected for detection: rs4986771, rs138898320, rs4987241, and rs2278415. Among them, rs4986771, rs4987241, rs2278415 had missense mutations, while rs138898320 had synonymous mutation (Table 2).

**Table 1:** *ZBRK1/ZNF350* gene exon mutations and function prediction (case group)

SNP No	Nucleotide change	SNP site	Location	Amino acid change	Mutation type	SIFT protein change	SIFT Prediction score
1	c.105 T > C	rs4986773	EXON3	p.Asp35Asp	S	No effect	-
2	c.111G > A	rs4987241	EXON3	p.Met37Ile	M	Impairment	0.00
3	c.197 T > C	rs2278420	EXON4	p.Leu66Pro	M	Neutral	0.47
4	c.708 T > C	rs4988334	EXON5	p.Cys236Cys	S	No effect	-
5	c.1119C > A	rs3764538	EXON5	p.Pro373Pro	S	No effect	-
6	c.1503A > T	rs2278415	EXON5	p.Arg501Ser	M	Impairment	0.04
7	c.1155A > C	rs4986772	EXON5	p.Thr385Thr	S	No effect	-
8	c.1414 T > C	rs4986771	EXON5	p.Ser472Pro	M	Neutral	0.10
9	c.100C > T	rs138898320	EXON3	p.Arg34Trp	M	Neutral	0.09

The SIFT prediction score < 0.05 indicated that it affected the protein function. S, synonymous mutation; and M, missense mutation

**Table 2:** *ZBRK1/ZNF350* gene exon site mutations (control group)

SNP No	SNP site	Location	Nucleotide change	Amino acid change	Mutation type
1	rs4986771	EXON5	c.1414 T > C	p.Ser472Pro	M
2	rs138898320	EXON3	c.100C > T	p.Arg34Trp	S
3	rs4987241	EXON13	c. 111G > A	p.Met37Ile	M
4	rs2278415	EXON5	c.1503A > T	p.Arg501Ser	M

### General information of case and control groups

Analysis of the general information of the case and control groups showed that, there were no statistically significant differences in BMI, age of menarche, age of first delivery, or times of pregnancies between the case and control groups (all *P* > 0.05). However, significant difference was observed in the frequency distribution of breast cancer family history between the case and control groups (*P* = 0.006) (Table 3).

**Table 3:** General analysis of the case and control groups

	Case group (N = 80)	Control group (N = 240)	*P*
BMI	23.02 ± 3.92	23.02 ± 3.65	0.963
Menarche age, yr	13.28 ± 1.59	12.98 ± 1.28	0.099
Age of first delivery, yr	27.25 ± 3.77	27.33 ± 3.24	0.864
Number of pregnancy			
0–1	34 (42.5)	98 (40.8)	
≥ 2	46 (57.5)	142 (59.2)	0.793
Family history of cancer			
Yes	18 (22.5)	24 (10.0)	
No	62 (77.5)	216 (90.0)	0.006

The χ^2^ test was used to compare counting data (expressed as n(%)) between the case and control groups, and the t-test was used to compare measurement data (represented by mean ± SD) between the case and control groups

### Distribution of ZBRK1/ZNF350 gene exon genotype in case and control groups

Analysis of *ZBRK1/ZNF350* gene exon genotype distribution showed that, the CT genotype frequency of rs138898320 locus was significantly different between the case and control groups (χ^2^ = 11.33; *P* = 0.001). There was no statistically significant differences in the rs4986771, rs4987241, or rs2278415 genotype frequency distribution between the case and control groups (*P* > 0.05) (Table 4).

**Table 4:** *ZBRK1/ZNF350* gene exon genotype distribution in the case and control groups [n(%)]

Polymorphism	Case group (N = 80)	Control group (N = 240)	χ^2^	*P*
rs4986771				
TT	76 (95.00)	227 (94.58)		
CT	4 (5.00)	13 (5.42)	0.00	1.000
rs4987241				
GG	75 (93.75)	222 (92.50)		
GA	5 (6.25)	18 (7.50)	0.14	0.708
rs138898320				
CC	72 (90.00)	237 (98.75)		
CT	8 (10.00)	3 (1.25)	11.33	0.001
rs2278415				
AA	49 (61.25)	131 (54.58)		
AT	28 (35.00)	93 (38.75)	0.633	0.426
TT	3 (3.75)	16 (6.67)	0.647	0.412
AT + TT	31 (38.75)	109 (45.42)	1.084	0.298

Note: The χ^2^ test was used to compare counting data (expressed as n(%)) between the case and control groups, and the t-test was used to compare measurement data (represented by mean ± SD) between the case and control groups

### Logistic regression analysis of ZBRK1/ZNF350 gene exon genotype and breast cancer susceptibility

Our results showed that, compared with the GG wild genotype carrier at the rs4987241 site, the risk of breast cancer was increased by 21.6% (OR = 1.216; 95% CI 0.436–3.389) for the GA carriers at the rs4987241 site. However, there was no statistical significance (*P* = 0.708). Moreover, compared with AA wild genotype carriers at the rs2278415 site, the risk of breast cancer for AT genotype carriers was increased by 17.5% (OR = 1.175; 95% CI 0.693–1.992), while the risk of breast cancer was increased by 83% for the TT genotype carriers (OR = 1.833; 95% CI 0.52–6.463). However, no statistical significance was observed (*P* = 0.549 and *P* = 0.346, respectively). Furthermore, the risk of breast cancer was reduced by 67.8% (OR = 0.322; 95% CI, 0.079–1.319) for the CT genotype carriers than that for the TT genotype carriers at rs4986771 (*P* = 0.115) (Table 5). In addition, compared with the carriers of CC wild genotype at rs138898320, the risk of breast cancer was significantly reduced by 88.3% in the CT mutation genotype carriers (OR = 0.117; 95% CI 0.030–0.455) (*P* = 0.002) (Table 6). Moreover, family history stratification was performed. In the stratification without family history, compared with the carriers of CC wild genotype at rs138898320 site, significant difference was observed in the carriers of CT mutation genotype (OR = 0.107; 95% CI 0.020–0.564) (*P* = 0.008). On the other hand, in the stratification with family history, there was no significant difference in the variation of rs138898320 (OR = 0.185; 95% CI 0.018–1.941) (*P* = 0.159) (Table 7).

**Table 5:** Logistic regression analysis of *ZBRK1/ZNF350* gene exon genotype and breast cancer susceptibility

Polymorphism	β	SE (β)	Wals	*P*	OR	95%CI
rs4986771						
TT					1.000	
CT	-1.133	0.719	2.481	0.115	0.322	0.079–1.319
rs4987241						
GG					1.000	
GA	0.196	0.523	0.140	0.708	1.216	0.436–3.389
rs138898320						
CC					1.000	
CT	-2.172	0.690	9.904	0.002	0.114	0.029–0.441
rs2278415						
AA					1.000	
AT	0.161	0.269	0.358	0.549	1.175	0.693–1.992
TT	0.606	0.643	0.889	0.346	1.833	0.520–6.463
AT + TT	0.274	0.264	1.081	0.299	1.315	0.785–2.205

**Table 6:** Logistic regression analysis of *ZBRK1/ZNF350* gene exon genotype and breast cancer susceptibility after correction

Polymorphism	β	SE (β)	Wals	*P*	OR	95%CI
rs4986771						
TT					1.000	
CT	-0.416	0.604	0.474	0.491	0.660	0.202–2.154
rs4987241						
GG					1.000	
GA	0.239	0.599	1.590	0.690	1.270	0.393–4.105
rs138898320						
CC					1.000	
CT	2.026	0.710	8.346	0.004	7.581	1.918–26.963
rs2278415						
AA					1.000	
AT	-0.095	0.281	0.116	0.734	0.909	0.524–1.576
TT	-0.747	0.656	1.300	0.254	0.474	0.131–1.712
AT + TT	-0.181	0.270	0.451	0.502	0.834	0.491–1.416

**Table 7:** Logistic regression analysis of rs138898320 locus and breast cancer susceptibility after family history stratification

Family history	Polymorphism	β	SE (β)	Wals	*P*	OR	95%CI
Yes	rs138898320						
	CC					1.000	
	CT	1.056	1.267	0.694	0.405	2.875	0.240–34.462
No	rs138898320						
	CC					1.000	
	CT	2.439	0.830	8.633	0.003	11.464	2.253–58.344

## Discussion

*ZBRK1/ZNF350* is located on chromosome 19 GRCh37.p2; 52,490,079–52,467,593 [18], covering about 10 kb and containing 4 exons [13], which encodes a protein of 60-kDa [15]. This protein contains the highly conserved KRAB domain at the NH2 end, 8 continuous C2H2 zinc finger motifs at the COOH end, and the CTRD domain. ZBRK1/ZNF350 protein inhibits transcription through CTRD with independent DNA binding [19]. The N-terminal KRAB domain of ZBRK1/ZNF350 protein binds to the repressor protein to form a transcriptional repression domain [20]. ZBRK1/ZNF350 protein is related to the occurrence of various human tumors [21]. Garcia-Closas et al. [22] have conducted a meta-analysis based on the population of the United States (3368 cases and 2880 controls) and Poland (1995 cases and 2296 controls). They have found that there may be a weak link between the *ZBRK1/ZNF350* rs4986771 locus and the risk of breast cancer. In the American population, compared to the wild-type homozygous genotype, heterozygotes at the *ZBRK1/ZNF350* rs4986771 locus would suffer from increased risk of breast cancer, and the same genotype does not have significantly increased risk in the Polish population. A meta-analysis has showed that the rs4986771 gene mutation may have a protective effect on breast cancer [23]. However, in this study, our results showed that there was no statistically significant difference in the rs4986771 genotype frequency distribution between the case and control groups. The main reason for the difference between our conclusions and the meta-analysis [23] may be the sample size and study design. Their study was a meta-analysis performed on the population from the US (3368 cases and 2880 controls) and Poland (1995 cases and 2296 controls) with large sample size. However, the sample size in our original article was small. Further studies are needed.

In this study, through the direct sequencing of the exon region of the *ZBRK1/ZNF350* gene in 80 cases of early-onset (30–40 years old) breast cancer who were admitted to our hospital, a total of 9 sequence variants were detected. Garcia et al. [11] have performed the *ZBRK1/ZNF350* gene sequencing on patients with primary breast cancer and have found 9 polymorphic sites, among which 7 sites were consistent with the sites found herein, namely rs2278420, rs2278415, rs4986771, rs4986773, rs4988334, rs3764538, and rs4986772. The SIFT software was used to predict the protein function, and our results showed that the sequence variants rs4987241 and rs2278415 in the breast cancer patients were harmful mutations, and one of the patients was the carrier of c.111G > A in rs4987241 aged 34 years old, who suffered from bilateral breast cancers (axillary lymph node metastasis at both sides, 3/22 right breast and 1/26 left breast; the left breast was triple negative breast cancer). Another breast cancer patient also suffered from bilateral breast cancer, who was aged 37 years old, and both breasts were triple-negative breast cancers. However, in this study, there were no significant differences in the genotype frequencies of rs4987241 and rs2278415 distribution between the case and control groups. However, a meta-analysis has shown that the risk of breast cancer is increased for the rs2278415 gene mutation [23]. This phenomenon may be related to the sample size, and this site worth further investigation with enlarged sample sizes.

Polymorphism refers to a DNA sequence polymorphism caused by the substitution, insertion or deletion of a single nucleotide at the genome level. It is a common type of human heritable variation, accounting for more than 90% of known polymorphisms. When the minimum mutation frequency is greater than 1%, it would be called polymorphism. As shown in Table 4, the mutation rate of a single base of the rs138898320 allele was 3/480, and the allele frequency was less than 1%. Therefore, this locus may be a rare mutation rather than a polymorphism. In this study, it is worth noting that, compared with the carriers of CC wild genotype at rs138898320, the risk of breast cancer was reduced by 88.3% in carriers of CT mutant genotype, with statistically significant differences. In the stratification without family history, compared with the carriers of CC wild genotype at rs138898320, the CT mutation genotype carriers had protective effects on breast cancer, with statistically significant differences. However, in the stratification with family history, there was no significant difference in the variation of rs138898320 of *ZBRK1/ZNF350* gene polymorphism.

Menstruation and fertility are important components of physiological reproductive factors. It has been shown that the menarche and age of first birth are related to the incidence of breast cancer. Early age of menarche and late age of first birth are important risk factors for breast cancer. In this study, our results showed that there was no statistically significant difference in BMI, age of menarche, age of first birth, or times of pregnancies between the case and control groups, and there was significant differences between the case and control groups with the breast cancer family.

## Conclusion

In conclusion, the nine sequence variants found by the direct sequencing of the *ZBRK1/ZNF350* gene exon region in this study were mostly consistent with previous findings, still with however some differences. To further clarify the relationship between the *ZBRK1/ZNF350* gene SNPs and breast cancer susceptibility, based on our findings herein, further in-depth studies with enlarged sample sizes concerning different populations are still needed.

## Data Availability

The reference sequence used was from the gene database of NCBI (*ZNF350* zinc finger protein 350 [Homo sapiens (human)]; Gene ID: 59,348; https://www.ncbi.nlm.nih.gov/gene/59348). The datasets generated and/or analysed during the current study are deposited in European Variation Archive (EVA) under analysis accession number "ERZ1691763" (https://www.ebi.ac.uk/ena/browser/view/ERZ1691763).

## Abbreviations

BRCA1: Breast cancer susceptibility gene 1
BRCA2: Breast cancer susceptibility gene 2
ZBRK1: Zinc finger and BRCA1-interacting protein with KRAB domain-1
ZNF350: Zinc-finger protein 350
